# Operation for Ovarian Tumor, by Prof. Meeker, of La Porte, Indiana

**Published:** 1859-06

**Authors:** Charles Brackett

**Affiliations:** Rochester, Indiana


ARTICLE III.

### OPERATION FOR OVARIAN TUMOR, BY PROF. MEEKER, OF LAPORTE, IND.—REMOVAL OF SEQUESTRUM, BY CHARLES BRACKETT, OF ROCHESTER, IND.

A short time since, I read a letter from Prof. D. Meeker, of Laporte, who says he was the surgeon who attended the woman of Tippecanoe, Marshall county, Ind., and that he is perfectly satisfied that her shoulder was fully—perfectly reduced, at the time he undertook the case. In the February number of the *Chicago Medical Journal*, page 89, I reported her case as "dislocation of the humerus, of eleven weeks;" in which I stated that, "at Laporte, when reduction was again undertaken by the good surgeons, assisted by Jarvis' adjuster, this operation also failed. Some seven or eight weeks after which, I saw her, and reduced the shoulder.

In justice to Professor Meeker, I make this statement. The Professor says "that the shoulder was reduced at the time I made the attempt at Laporte. I think that I was not mistaken about it at the time I was assisted by Dr. Bruise."

On page 89 of the February number of the *Journal*, and sixteenth line, it should read, Dr. Southall, of Tippecanoe, Ind., for "Dr. South, all of Tippecanoe, Iowa."

The following case of ovariotomy, by Prof. Meeker, at which I assisted, by his consent, I submit for publication:

Mrs. Fennimore, aged about forty, had, for the past two years, an ovarian tumor. After the tumor had acquired a diameter of about eight inches, she became pregnant. She first consulted me as to the propriety of inducing abortion, as she had been informed that gestation could not be fully accomplished, except with a fatal result. I assured her that it could; that from the situation of the tumor, the gravid uterus, enlarging, would mould itself into, and produce absorption of, the tumor. With this assurance, she became quieted; went a full term, with a tranquil mind, and was safely delivered of a healthy, full-grown child. At that time the tumor could be distinctly felt; convex on side next the womb, and materially diminished in volume. About September 1st, 1858, on account of retention of urine, from mechanical pressure (the tumor, for a year after her confinement, having grown wonderfully fast), and from other threatening symptoms, she concluded to have it removed by an operation, and chose Prof. Meeker, of Laporte, as her operator. Accordingly, on the 11th of September, I, in company with Mr. Palmer, Dr. Gould, and Dr. R. A. Diier, of Union, Marshall county, and Dr. Wm. Demoss, met Prof. Meeker at the residence of Mr. Fennimore, sixteen miles west of Rochester.

From the size of the tumor, it was necessary to make the abdominal incision from the xiphoid cartilage to the pubis. On turning out the tumor, it was found to be adherent to the greater part of the right side of the uterus, including the fallopian tube to the bladder, and low down the iliac region, closely investing the right primitive iliac artery, and the whole right iliac region posteriorly; covered externally with a close net-work of large veins, or venous sinuses, presenting a mass of such vascularity, as that, probably, almost immediate death would have followed even a small incision into the surface of the tumor.

The Professor then punctured the tumor, where it appeared least vascular, with a trocar. This was followed by a welling out of the blood in a stream the size of the trocar, and could only be restrained by passing a double thread through the peritoneal surface transversely to the puncture, and tying both ways. A second puncture was made over a portion of the tumor apparently filled with yellowish serum. This was followed by a like gush of blood, and but a small quantity of serum. The bleeding was here restrained in like manner, by the needle and ligature. With great difficulty the tumor and intestines were returned into the abdominal cavity, and the wound closed by sutures, adhesive strips, and bandages. I stayed with the patient most of the time for a couple of days, and, for a week, kept her quiet with morphine as often as necessary, to produce the desired effect. With occasional laxatives, as occasion demanded, she became quite comfortable, and, in fact, had no severe symptoms at any time during the operation, except a good deal of prostration. She improved rapidly for a week. The wound healed; but on the 30th of November following, eleven weeks and three days after the operation, she died. A post-mortem examination, made by Dr. Diier, revealed the fact that the *side of the tumor which had been punctured, was mostly absorbed*, and the remainder was transformed, or degenerated, into a substance resembling adipocire. I have not the notes of the necropsy, which were promised me by the Doctor, so cannot now give a full account of post-mortem appearances.

Should I ever have a case similar, or one where, from extensive adhesion, the tumor could not be removed, I should pass large setons, traversing the tumor in different directions, and would expect, in a patient as tenacious of life as was Mrs. Fennimore, a perfect cure of the disease by suppression or absorption, one, or both. I am led to this conclusion from the effect of the sutures made through a small extent of this tumor, and from the well known good effects of a seton passed through some external tumors, which it is not advisable, or desirable, to remove with the knife.

### REMOVAL OF SEQUESTRUM FROM FEMUR.

Wm. Culver, aged about forty, scrofulous diathesis, twenty years lame from an attack of what was called white swelling; since which time, knee joint anchylosed; suppurative discharge from parts above the joint. Now, February 14th, 1859, confined to one fistula on inner aspect of thigh, two and a half inches above knee joint; in which fistula, at the bottom, can be felt with a probe, dead bone, probably a sequestrum. Assisted by Dr. Gould and Jonas Myers, I laid open the integument to the femur by an incision three inches long, following the course of the fistula—then, could feel the loose bone through a small opening, sufficiently large to admit the point of the index finger; sides of this opening seemed cartilaginous; enlarged the opening till, with my forceps, I drew out a piece of bone three inches in length, with irregularly serrated edges. Venous hemorrhage profuse; wound closed, and healed rapidly, leaving more mobility to the parts than before the operation. From time to time, to this date, May 17th, 1859, after some soreness about the leg, suppuration takes place, and small pieces of carious bone, similar to the edges of the piece removed, of the size of a pin's head, are discharged. These, I think, are only pieces broken off from the main piece, on its removal from its bed in the femur. I think that the leg will, ultimately, become well, as, at the time of the operation, all parts of bone surrounding the sequestrum were covered with healthy periosteum, perfectly sound, as far as I could know.

